# Regulation of the transcriptome by ER stress: non-canonical mechanisms and physiological consequences

**DOI:** 10.3389/fgene.2013.00256

**Published:** 2013-12-02

**Authors:** Angela M. Arensdorf, Danilo Diedrichs, D. Thomas Rutkowski

**Affiliations:** ^1^Department of Anatomy and Cell Biology, University of Iowa Carver College of MedicineIowa City, IA, USA; ^2^Department of Mathematics and Computer Science, Wheaton CollegeWheaton, IL, USA; ^3^Department of Internal Medicine, University of Iowa Carver College of MedicineIowa City, IA, USA

**Keywords:** ER stress, unfolded protein response (UPR), mRNA stability, Transcription Factors, gene regulatory networks (GRN)

## Abstract

The mammalian unfolded protein response (UPR) is propagated by three ER-resident transmembrane proteins, each of which initiates a signaling cascade that ultimately culminates in production of a transcriptional activator. The UPR was originally characterized as a pathway for upregulating ER chaperones, and a comprehensive body of subsequent work has shown that protein synthesis, folding, oxidation, trafficking, and degradation are all transcriptionally enhanced by the UPR. However, the global reach of the UPR extends to genes involved in diverse physiological processes having seemingly little to do with ER protein folding, and this includes a substantial number of mRNAs that are suppressed by stress rather than stimulated. Through multiple non-canonical mechanisms emanating from each of the UPR pathways, the cell dynamically regulates transcription and mRNA degradation. Here we highlight these mechanisms and their increasingly appreciated impact on physiological processes.

## Introduction

The ER is best known as the gateway to the secretory pathway. As the site of synthesis for nascent secretory proteins and resident lumenal and transmembrane proteins of the endomembrane system, the ER shepherds the folding, oxidation, modification, and assembly of approximately one-third of the cellular proteome—or more in cell types specialized for protein secretion such as antibody-secreting plasma B lymphocytes or endocrine or exocrine cells (Huh et al., 2003; Tagliavacca et al., 2003). As such, the ER is replete with chaperones, cochaperones, oxidases, and thiol isomerases to facilitate protein folding, and utilizes an elaborate quality control system to recognize terminally misfolded proteins and purge them from the ER for degradation (Araki and Nagata, 2012). With this system in place, the low level of protein misfolding that arises because of the inherent error rate in the process can presumably be managed. However, the quality control machinery can be overwhelmed either by an overload of nascent client proteins or by any exogenous disruption to the protein folding and trafficking system—so-called “ER stress” (Ron and Walter, 2007). The consequences of a pervasive defect in ER protein folding can be grave for both the cell and the organism. A cell with overwhelmed ER quality control machinery will, at best, fail to maintain secretory pathway integrity and to effectively sense and respond to the extracellular milieu. At worst, accumulated misfolded proteins might seed the formation of toxic protein aggregates (Matus et al., 2011). Thus, the cell has in the UPR a signal transduction system that augments the protein folding capacity of the ER. While the UPR improves ER function by several short-term mechanisms, it ultimately culminates in gene regulation for longer-lasting enhancement of the ER folding environment. Classically, this regulation constitutes a self-contained system in which ER stress leads to transcriptional induction of genes encoding ER chaperones and other proteins that grease the wheels of secretory pathway function, thereby alleviating ER stress and shutting the response off (Travers et al., 2000). However, it is now becoming clear that the UPR is much more deeply entwined in cellular physiology than this simple view would suggest. In mammals, it regulates genes involved in a number of cellular processes that have little on their face to do with ER function, including metabolism and inflammation (Fu et al., 2012; Garg et al., 2012). The regulation of many of these genes cannot be explained by the canonical mechanisms of UPR signaling. The aim of this review is to highlight emerging concepts in the non-canonical regulation of mRNA expression by the UPR. Rather than providing an exhaustive account of all possible means by which mRNA abundance might be controlled by the UPR, here we describe the general principles by which such regulation can occur and provide illustrative examples that emphasize the diverse physiological consequences of such pathways.

## The canonical UPR

The idea that there must be a signal transduction pathway emanating from the ER first emerged from the observation that expression of misfolded influenza hemagglutinin (HA) in mammalian cultured cells led to upregulation of the ER chaperones *Bip* (aka GRP78, the product of the *Hspa5* gene) and GRP94 (the product of the *Hsp90b1* gene) (Kozutsumi et al., 1988). This finding allowed previous reports of *Bip* and *Grp94* induction in response to chemical perturbants (Drummond et al., 1987; Kim and Lee, 1987) to be tied specifically to disrupted ER protein folding. The general applicability of the phenomenon was extended to other misfolded ER client proteins (Dorner et al., 1989), and to the upregulation of ER oxidases and thiol isomerases as well (Dorner et al., 1990). As with many fundamental cellular processes, an analogous response was soon discovered in yeast (Normington et al., 1989), and a *cis*-acting unfolded protein response element (UPRE) within the yeast *Bip* (aka *KAR2*) promoter was discovered (Mori et al., 1992; Kohno et al., 1993). The ER stress-responsiveness of *KAR2* served as the springboard for a classic series of genetic and biochemical studies describing the mechanistic basis of what had become known as the unfolded protein response (Cox et al., 1993; Mori et al., 1993; Cox and Walter, 1996; Sidrauski et al., 1996; Sidrauski and Walter, 1997). Together, these studies identified Ire1p (for Inositol-requiring enzyme) as an ER-resident transmembrane kinase that became autophosphorylated during ER stress, activating a cytosolic endoribonuclease activity that catalyzed the removal of an inhibitory intron from *HAC1* mRNA. This splicing event allowed the mRNA to be translated into the Hac1p transcription factor, which subsequently bound to the *KAR2 UPRE* and stimulated *KAR2* transcription. A survey of the breadth of UPR targets (Travers et al., 2000) took advantage of then-new microrarray technology, and revealed two fundamental features of the yeast UPR that have heavily influenced the subsequent portrayal of the response in both yeast and mammals: (1) It is predominantly an inductive response, with a bias for upregulated genes; and (2) its scope is not limited solely to ER chaperones and phospholipid synthesis enzymes, as had been originally thought, but encompasses genes involved in other aspects of secretory pathway function, including ER-associated protein degradation (ERAD), vesicular trafficking, and protein translocation into the ER, among other processes. Thus, even though the response was more expansive than anticipated, it could still be considered as a discrete transcriptional program designed to upregulate the expression of genes with a common *cis*-element, all of which influenced ER protein folding either directly or indirectly.

Once Ire1p was described in yeast, its mammalian homologs (IRE1α and an intestine-specific paralog IRE1β) were discovered (Tirasophon et al., 1998; Wang et al., 1998), as was a gene encoding a transmembrane protein with a lumenal domain homologous to IRE1α but a cytosolic aspect homologous to other kinases such as PKR that phosphorylate the translation initiation factor eIF2α. The protein encoded by this gene was named PERK (PKR-like ER kinase) (Shi et al., 1998; Harding et al., 1999). And, much as the *UPRE* had been instrumental in identification of Ire1p in yeast, so also a mammalian ER stress response element (*ERSE*) in the promoters of *Bip* and *Grp94* was characterized and used to identify ATF6 (of the activating transcription factor family) as another ER-resident stress sensor (Yoshida et al., 1998; Haze et al., 1999). All three pathways are conserved throughout metazoa, but the PERK and ATF6 pathways appear to assume more modest roles in invertebrates such as flies and worms (Shen et al., 2001; Ryoo and Steller, 2007).

As for yeast Ire1p, activation of each of the three mammalian UPR pathways culminates in production of a transcriptional activator and attendant rearrangement of chromatin structure (Baumeister et al., 2005; Donati et al., 2006; Gal-Yam et al., 2006) and recruitment of RNA Polymerase II (Sela et al., 2012) to stimulate gene transcription. These pathways have been reviewed exhaustively elsewhere (Schröder and Kaufman, 2005) and so will only be described briefly here. Mammalian IRE1α is activated by autophosphorylation and catalyzes the splicing of *Xbp1* mRNA, resulting in excision of a 26-base intron and thus allowing in-frame translation of the downstream transcriptional activation domain of XBP1 (Yoshida et al., 2001a; Lee et al., 2002). Translocation and activity of the bZIP protein produced from spliced *Xbp1* mRNA are regulated by the protein produced by the unspliced mRNA (Lee et al., 2003a; Tirosh et al., 2006), by the regulatory subunits of PI3 Kinase (Park et al., 2010; Winnay et al., 2010), and by acetylation (Wang et al., 2011). In the nucleus, XBP1 binds to *UPRE* sequences (distinct from yeast *UPRE* elements) in, among others, the promoters of genes encoding ERAD factors (Yoshida et al., 2001b; Lee et al., 2003b; Yoshida et al., 2003; Yamamoto et al., 2007). XBP1 can also bind to additional non-*UPRE* sequences and regulate genes involved in phospholipid biosynthesis (Sriburi et al., 2007), lipogenesis (Lee et al., 2008) and myogenic differentiation (Acosta-Alvear et al., 2007). Mice lacking XBP1 die prenatally due to liver defects (Reimold et al., 2000), and mice lacking IRE1α die even earlier with both liver and lymphocyte differentiation defects (Zhang et al., 2005) that might be secondary to placental failure (Iwawaki et al., 2009). Liver-specific rescue of XBP1 deficiency only postpones death into the neonatal period, when dysmorphogenesis of the exocrine pancreas leads to digestive failure (Lee et al., 2005).

PERK activation and autophosphorylation lead to eIF2α phosphorylation which, while having the immediate (and transient) effect of inhibiting the translation of most mRNAs, stimulates translation of the *Atf4* mRNA due to the presence of upstream open reading frames (uORFs) in the *Atf4* 5' UTR (Lu et al., 2004; Vattem and Wek, 2004). This effect is a consequence of the inefficient ribosome assembly brought on by eIF2α phosphorylation, which allows certain mRNAs—2 to 8 percent by one estimate—to be translationally stimulated rather than inhibited, based on the presence of uORFs (Ventoso et al., 2012; Barbosa et al., 2013). Like XBP1, ATF4 is a bZIP transcription factor; it binds to amino acid response elements (*AARE*s) in target gene promoters. eIF2α can be phosphorylated by other kinases in response to various cellular stresses, and this pathway of signal transduction is known as the integrated stress response (ISR) (Harding et al., 2003). *Perk*^−/−^ mice develop progressive postnatal diabetes and exocrine pancreas disruption (Harding et al., 2001).

Finally, ATF6 is an ER-localized transmembrane transcription factor. ER stress releases it from the ER to the Golgi, where it is cleaved by regulated intramembrane proteolysis (RIP) to liberate the transcriptionally active cytosolic domain—itself a bZIP family member—that dimerizes with the constitutive factors NFY and YY1 and binds to *ERSE* and *ERSE-II* sequences in target genes (Li et al., 2000; Ye et al., 2000; Yoshida et al., 2000; Kokame et al., 2001; Baumeister et al., 2005). ATF6 can also heterodimerize with XBP1 on *UPRE* sites (Yamamoto et al., 2007); indeed, the mammalian *UPRE* was first identified by virtue of its binding by ATF6 (Wang et al., 2000). Mice lacking ATF6 are overtly normal (Wu et al., 2007; Yamamoto et al., 2007), and no basal phenotype has yet been reported, although this surprising absence might be due to functional redundancy between ATF6 and its paralog ATF6β (discussed in more detail later).

**Figure 1:**
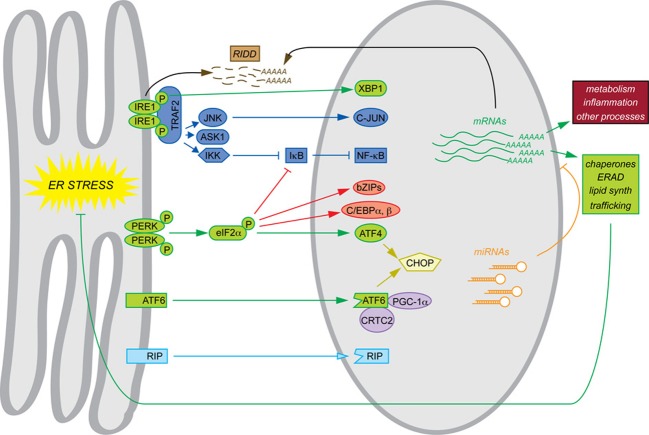
**Canonical and non-canonical pathways of mRNA regulation**. Examples of each of the pathways of mRNA regulation discussed in this review are shown. The canonical UPR pathways are shown in green. Also depicted are transltional regulation (red), scaffolding (dark blue), transcriptional cascades (yellow), cofactor titration (purple), alternate RIP substrates (light blue), RIDD (brown), and miRNAs (orange). Together, these processes result in the regulation not only of ER protein folding function (green) but also other cellular processes such as metabolism and inflammation (maroon).

The phenotypes of mice with constitutive deletions of UPR components mesh with the narrative of the UPR as a self-contained program for maintaining ER protein folding homeostasis. Where basal phenotypes are evident, disrupted cell types in the various animals show evidence of grossly altered ER structure and impaired secretory pathway function. In addition, constitutive deletion of *Xbp1*, *Atf4*, and *Atf6* allowed the transcriptional programs downstream of each to be examined by microarray in the most convenient cell type, mouse embryonic fibroblasts (MEFs)—the cell type in which most of the basic pathways of the UPR were elucidated. Similarly to yeast, MEFs respond to ER stress with an upregulation of genes encoding ER chaperones and cochaperones, ERAD factors, lipid synthesis enzymes, and other proteins of importance to secretory pathway function and general protein biosynthesis. Subsets of these genes were found to depend on each of the three UPR-regulated bZIP transcription factors. Although there is considerable overlap in the sets of genes regulated by these factors, to a first approximation ATF4 coordinates the upregulation of genes involved in protein anabolism and redox defense (Harding et al., 2003), XBP1 appears to regulate ERAD (Lee et al., 2003b), and ATF6 contributes to upregulation of chaperones and ERAD factors (Wu et al., 2007; Adachi et al., 2008). For the purposes of this review, we shall refer to this mechanistic framework, culminating in production of XBP1, ATF4, and ATF6—along with the direct actions of these factors on target genes—as the canonical UPR, and it is indicated in green in the accompanying figure to highlight the non-canonical mechanisms that are the focus of this review (Figure 1).

A deeper look at these microarray studies reveals that the transcriptional output of the UPR is not so simple and self-contained. The emphasizing of the UPR as a program for transcriptional induction meant that the downregulated genes were not characterized in each of these analyses; yet between one quarter and one half of mRNAs regulated by ER stress are actually suppressed, with few mechanisms to account for them. In contrast to upregulated genes, the suppressed genes cluster among a number of cellular processes having apparently little to do with ER protein folding (Arensdorf and Rutkowski, 2013). Further, even among genes upregulated by ER stress, only a relatively small number can be tied definitively to ATF4, XBP1, and/or ATF6. The mechanisms responsible for regulation of the majority of genes during ER stress even in a presumptively “generic” cell type such as the MEF are not understood, and quite possibly fall outside the scope of the canonical UPR.

## Temporal dynamics of mRNA regulation

There are four ways in which mRNA abundance might be regulated by the UPR: stimulation or inhibition of transcription, and enhancement or compromise of mRNA stability. The historical view of the UPR as a program for upregulation of ER chaperones has shone the most attention on the first mechanism, but the other three contribute as well. Indeed, they probably collectively contribute to a substantial fraction of the observed changes in mRNA expression upon ER stress, or perhaps even the majority of it. The mechanism by which an mRNA is regulated has implications for the timing and persistence of that event and, by extension, the window of time during which the protein product translated from that mRNA is able to influence cellular function.

**Figure 2:**
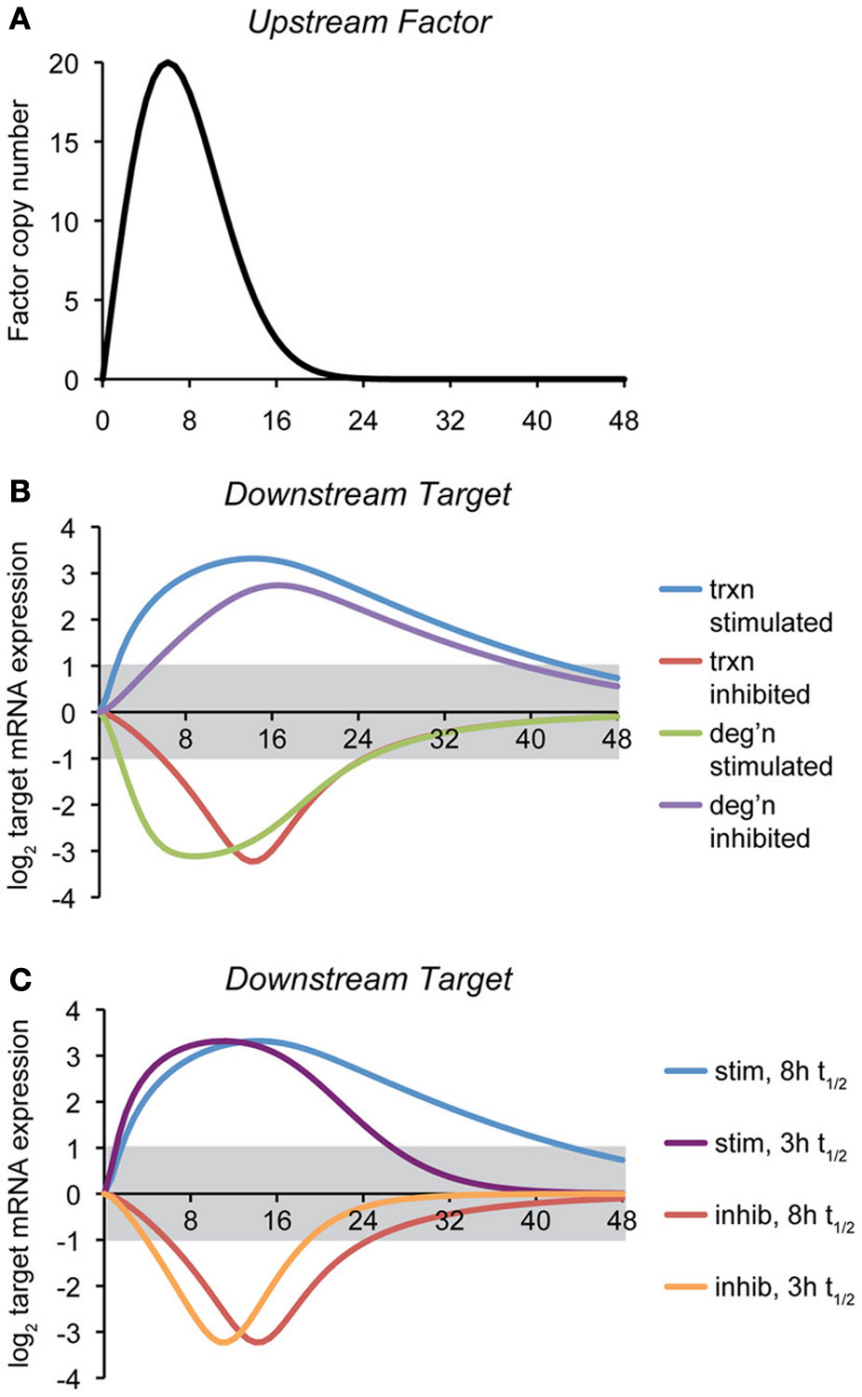
**Temporal dynamics of mRNA regulation by different mechanisms. (A)** A computational model was created in which the expression of a downstream target mRNA is directly controlled by the induction of an upstream factor, the expression of which is shown. The behavior of this factor is modeled loosely off of the dynamics of ATF4, ATF6, and XBP1 upon a level of ER stress to which cells can successfully adapt (Rutkowski et al., 2006). **(B)** Expression of the downstream target mRNA was modeled based on the upstream factor either stimulating or inhibiting transcription, or stimulating or inhibiting degradation, under conditions where maximal expression of the factor approaches the saturation level. For this simulation, the intrinsic (i.e., unregulated) half-life of target mRNA was chosen to be 8 h, and the parameters were then chosen to elicit 10-fold maximal regulation in expression, and varying the rate- and affinity-constants did not change the essential behavior of the model. The gray region indicates changes in mRNA levels that are less than two-fold (the threshold most frequently used to identify regulated genes in microarray experiments). The various curves illustrate two principles: (1) the window of time in which the expression of an mRNA will appear to be regulated (based on the two-fold criterion) depends on the mechanism of regulation, and (2) downregulation of mRNA either by inhibition of transcription or stimulation of degradation is necessarily shorter-lived than is upregulation, implying that changes in expression of downregulated genes might be easily overlooked. **(C)** The effect on transcriptional regulation of decreasing the half-life of the target mRNA form ~8 to ~3 h is shown. While the median half-life of cellular mRNA is 8–9 h, many of those encoding transcription factors have shorter half-lives (Schwanhausser et al., 2011). This includes that of *Chop*, which is itself transcriptionally regulated by the UPR.

To illustrate how the mode of regulation impacts the kinetics of mRNA expression, we provide here a simple computational model of a gene regulatory event, where the expression of a target gene is controlled by the expression of an upstream factor that can either stimulate transcription of the target gene, inhibit transcription, stimulate degradation of the target gene mRNA, or inhibit degradation (Figure 2A). The regulatory step is modeled as a non-cooperative interaction obeying simple Michaelis-Menten kinetics. Transcriptional effects depend upon the synthesis rate constant of the target transcript, the concentration of the upstream factor, and the affinity constant of that factor for its target gene. mRNA stability effects depend upon the same values, and also, as with any first-order decay process, on the concentration of the target mRNA itself. (The equations and parameters are given in the Supplemental Material).

The point of this exercise is to illustrate how—all other variables being held constant—the mechanism of mRNA regulation influences the rapidity with which the regulation is executed and its persistence. Thus, the hypothetical scenario shown here proposes that the action of the upstream factor has an effect on the target mRNA that results in its regulation (either up- or down-) by approximately ten-fold at its peak. With that stipulation, it then tests how the expression of an mRNA with an otherwise fixed rate of synthesis and rate of degradation behave in response to the expression of the upstream factor.

For this particular example, we modeled the response so that its peak effect was close to its saturation level, but the results were similar when the association/dissociation rate constants were varied over a wide range. This analysis reveals two salient features *that are largely independent of the actual parameters (rate constants and affinity constants) chosen*: First, mRNA levels can be more rapidly altered by stimulatory processes (of either transcription or degradation) than by inhibitory ones. Second, downregulated genes as a group return to basal expression levels more rapidly than do upregulated genes (Figure 2B). Further, the stability of the target mRNA influences the window of time when its expression is regulated, with shorter half-lives causing mRNAs to more directly mirror the expression of the upstream controlling factor (Figure 2C). In other words, while factors intrinsic to an mRNA (its synthesis and degradation rate constants) influence the window of time in which the mRNA is expressed, “stretching” its expression curve to the right or left, the extrinsic mode of regulation determines whether the expression of an mRNA experiences a lag either as stress is first experienced (i.e., if transcription or degradation is inhibited) or when the response begins to resolve (i.e., when transcription or degradation is stimulated). These observations imply that varied mechanisms for mRNA regulation ensure that the UPR is a dynamically evolving amalgam of outputs rather than a single output that simply varies in intensity over time. Practically, they also imply that the timing of mRNA regulatory events will reveal important clues about the mechanisms responsible.

## Non-canonical pathways of transcriptional regulation

Non-canonical transcriptional outputs may arise as offshoots of the framework of the canonical UPR by three general mechanisms: (1) UPR pathway branching that results in the production of additional transcriptional regulators; (2) transcriptional cascades that expand the repertoire of targeted genes; and (3) alteration of the activity of constitutively expressed transcription factors through competition or cooperativity in the nucleus. In addition, UPR transcriptional output can be enhanced by the existence of parallel non-canonical stress-sensing pathways. Below, we provide examples of each of these modes of transcriptional regulation.

### Non-canonical regulation emanating from canonical pathways

The PERK and IRE1α cascades of the UPR in particular present multiple points at which additional signaling cascades could be initiated. First, both molecules are kinases, raising the possibility that other substrates exist. Both also self-associate during activation (Bertolotti et al., 2000), potentially forming stress-dependent scaffolds that can seed the assembly of signaling modules and that culminate in transcriptional regulation. In addition, the effects of eIF2α phosphorylation on protein synthesis potentially allow for the production of transcription factors in addition to ATF4.

#### Translational regulation

Phosphroylation of eIF2α initially suppresses the translation of 90% of cellular mRNA, which decreases to 50% within the first 3 h of stress (Ventoso et al., 2012). The effect of this suppression on the expression level of a given protein depends on the half-life of that protein and the duration of eIF2α phosphorylation, which is regulated by both constitutive (CreP) and inducible (GADD34) phosphatases (Jousse et al., 2003; Marciniak et al., 2004). Accordingly, the expression of proteins with short half-lives diminishes more rapidly than does that of long-lived proteins, as demonstrated initially for the cell cycle regulator Cyclin D1, the loss of which upon eIF2α phosphorylation leads to cell cycle arrest (Brewer et al., 1999). Translational inhibition offers the opportunity to transiently alter the composition of the transcription factor network based on the expression of both the factors themselves and upstream proteins that regulate transcriptional cascades. While transcription factors as a class tend to have short half-lives, the range of their half-lives nonetheless spans an order of magnitude or more, meaning that inhibition of protein synthesis will have a more pronounced effect on the protein levels of some transcription factors than others (Schwanhausser et al., 2011, 2013).

UPR activation converges on inflammatory signaling in part through translation-dependent regulation of NF-κB. Members of the NF-κB/Rel family of transcription factors (i.e., NFKB1, NFKB2, c-REL, RELA, RELB) dimerize to form the NF-κ B transcriptional complex. The transcriptional activity of this complex is determined by its composition (Elsharkawy et al., 2010); and the complex regulates the transcription of genes involved in immunoregulation, growth regulation, inflammation, carcinogenesis and apoptosis (Hoesel and Schmid, 2013). NF-κB is sequestered in the cytoplasm by IκB; in order for NF-κB to be activated, the inhibitory subunit must be removed through phosphorylation and degradation (Ahn and Aggarwal, 2005).

PERK—through the phosphorylation of eIF2α—affects NF-κ B signaling by suppressing the translation of Iκ B family members (i.e., NFKBIE, NFKBIB), which have a shorter half-life than NF-κ B/Rel family members (Jiang et al., 2003; Deng et al., 2004). Thus, eIF2α phosphorylation decreases the amount of IκB relative to NF-κB. In addition, the translation of certain NF-κ B/Rel family members is suppressed during ER stress (i.e., REL, RELA, RELB) while others are not (i.e., NFKB1, NFKB2) (Ventoso et al., 2012), suggesting that eIF2α phosphorylation might not simply stimulate NF-κB activity, but might instead regulate the formation of specific NF-κ B complexes. The physiological significance of NF-κ B activation during ER stress is unknown; however, a number of NF-κ B target genes (Pahl, 1999) are found among ER stress-regulated genes, pointing to a contribution of NF-κB to UPR transcriptional output.

While NF-κB activity appears to be regulated passively by eIF2α phosphorylation through simple loss of an unstable inhibitor, translational control is also used to stimulate the translation of specific proteins, including several transcription factors beyond ATF4. Indeed, while the majority of mRNA translation is suppressed by eIF2α phosphorylation, the translation of ~2–8% of cellular transcripts is increased (Ventoso et al., 2012). Among the transcripts whose translation is stimulated are a number of transcription factors, including several among the bZIP family in addition to ATF4. These include ATF5 (Zhou et al., 2008), ATF3, CHOP, JUN, JUNB, FOS, FOSB, and CREB1 (Ventoso et al., 2012). How the translation of these transcripts is regulated during conditions of eIF2α phosphorylation is an area of active research; however, the presence of alternative uORFs within these transcripts is thought to dictate their translation during ER stress (Morris and Geballe, 2000).

In the case of the constitutively expressed transcription factors C/EBPα and C/EBPβ, translational control allows for the production of a truncated inhibitory form at the expense of a full-length activating form. C/EBP family members are bZIP transcription factors that participate in diverse physiological processes including differentiation, proliferation, metabolism, and inflammation (Ramji and Foka, 2002). Both α and β forms possess a cluster of potential translation initiation sites that are highly conserved among mammals. One of the start codons within this cluster is out of frame with respect to the others, and introduces a short open reading frame just upstream of the start codons that initiate translation of the full-length forms of α and β. The presence of this uORF is essential for translation of the truncated forms of both proteins, which is stimulated by eIF2α phosphorylation (Calkhoven et al., 2000; Wethmar et al., 2010). The truncated versions of both proteins have intact DNA binding domains but lack transactivation domains, making them potentially dominant-negative inhibitors of transcription (Descombes and Schibler, 1991).

Like *Atf4*, *Cebpa*, and *Cebpb* mRNAs both have uORFs that regulate the translation of alternate isoforms, but their expression appears to be controlled by a somewhat different mechanism. While the mechanism of C/EBPα translational regulation by eIF2α phosphorylation is still somewhat unclear, the expression of the long (aka Liver Activating Protein or LAP) and short (Liver Inhibiting Protein or LIP) forms of C/EBPβ are regulated by both eIF2α phosphorylation and dephosphorylation. Synthesis of the LIP form of C/EBPβ is translationally inhibited by eIF2α phosphorylation, with levels diminishing starkly because of its short half-life (Li et al., 2008). However, translational recovery promotes both increased synthesis and increased stability of the LIP form, causing its expression to predominate over that of LAP at later times after the induction of ER stress (Li et al., 2008; Arensdorf and Rutkowski, 2013). Thus, C/EBPβ, and probably C/EBPα as well, are more indirectly tied to eIF2α phosphorylation than is ATF4.

Translational regulation of both C/EBPα and C/EBPβ has been linked to diminished lipogenesis and improved glucose tolerance in mice fed a high-fat diet (Oyadomari et al., 2008). Mice with liver-specific overexpression of a constitutively active fragment of GADD34 that were raised on a high fat diet were resistant to weight gain and showed enhanced insulin sensitivity and reduced hepatic triglyceride accumulation. The reduced steady-state phosphorylation of eIF2α seen in these animals corresponded with lower expression of both C/EBPα and C/EBPβ (only the long forms of each were studied) and their downstream target genes, including the lipogenic mediator PPARγ. While a direct role for C/EBPα and β was not tested in GADD34-overexpressing mice, these findings illustrate a potentially significant physiological consequence of non-canonical ER stress-mediated transcription—namely, that eIF2α phosphorylation promotes hepatic lipid accumulation through enhanced or altered synthesis of C/EBPα and β.

C/EBPβ regulates inflammatory cascades in diverse cell types; it is alternatively named NF-IL6 based on its ability to regulate expression of the pro-inflammatory cytokine IL-6 (Akira et al., 1990). Preferential production of the LIP form of C/EBPβ during ER stress in cultured cells was found to result in transcriptional repression of a number of inflammatory genes, including IL4RA, which is an essential component of the IL-4 and IL-13 receptors (Arensdorf and Rutkowski, 2013). Most cell types express either the IL-4 or IL-13 receptor, and signaling through IL4RA stimulates pro-inflammatory processes as diverse as B cell proliferation, IgE class-switching, T_*H*_2 cell differentiation, smooth muscle contraction, mucus hypersecretion, eosinophil requirement, fibrotic deposition, and chemokine expression (Hershey, 2003; Wynn, 2003; Wills-Karp and Finkelman, 2008; Holgate, 2011). Remarkably, while ER stress suppressed IL-4/IL-13-dependent downstream signaling, this suppression was lost in *Cebpb*^−/−^ cells, indicating that the translational regulation of C/EBPβ can influence responsiveness to inflammatory signals. This finding suggests that UPR or ISR activation could influence the natural history of parasitic infections and allergic responses, both of which are accompanied by extensive IL-4 and IL-13 signaling (McKenzie, 2000).

#### Scaffolding

Both PERK and IRE1α self-associate during ER stress (Bertolotti et al., 2000). For yeast Ire1p, this self-association leads to the formation of oligomeric Ire1p clusters (Korennykh et al., 2009). While the dynamics of self-association for mammalian IRE1α and PERK are less understood, it seems likely that, here too, higher ordered multimeric complexes of both proteins form, resulting in the creation of potential stress-specific scaffold supports on the cytosolic face of the ER membrane (Li et al., 2010). IRE1α can seed the formation of cytosolic signaling modules, at least some of which culminate in transcriptional regulation (Hetz and Glimcher, 2009).

Among the proteins recruited to phosphorylated and oligomerized IRE1α is TNF receptor-associated factor 2 (TRAF2) (Urano et al., 2000). This association is thought to be required for activation of several kinases including IKK, JNK, ASK1, p38 MAPK, and ERK that contribute to cell fate decisions (i.e., survival vs. apoptosis) during ER stress (Urano et al., 2000; Nishitoh et al., 2002; Nguyen et al., 2004; Hu et al., 2006; Li et al., 2010; Tam et al., 2012). For IKK regulation, the subsequent activation of NF-κB (IKK phosphorylates IκB, leading to its degradation) was found to lead to induction of TNF-α expression (Hu et al., 2006). Likewise, JNK activation leads to phosphorylation of the bZIP transcription factor C-JUN (Urano et al., 2000), which has been implicated in ER stress-mediated regulation in cultured neurons of the gene encoding methylenetetrahydrofolate reductase, which participates in folate and homocysteine metabolism (Leclerc and Rozen, 2008), and of *Gpt1* and *Got1* in the liver, which encode the liver enzymes AST and ALT that are released from the liver upon damage (Josekutty et al., 2013). These examples notwithstanding, however, the contributions of IRE1α/TRAF2-dependent signaling to the sum of ER stress-mediated transcriptome control are poorly understood.

PERK also has the potential to recruit other molecules upon autophosphorylation and oligomerization, but there are fewer known parallel pathways arising from PERK activation than from IRE1α. PERK activation is necessary and sufficient for phosphorylation of the transcription factor NRF2 [Nuclear Factor (Erythroid-Derived 2)-Like 2] and can phosphorylate NRF2 *in vitro* (Cullinan et al., 2003). Cells lacking PERK or NRF2 do not effectively upregulate the NRF2-target genes *Gclc* or *Nqo1* upon ER stress (Cullinan and Diehl, 2004). PERK was also found to be required for activation of MAP kinase- and phospholipase C-dependent gene expression in response to ER calcium depletion (Liang et al., 2006a). However, as with IRE1α-dependent signaling modules, the global contribution of these pathways to mRNA regulation during ER stress is not clear.

### Transcriptional cascades

The output of the UPR is dramatically expanded by hierarchically arranged gene regulatory networks, in which the expression of subordinate transcription factors is targeted for regulation by the canonical UPR factors. Beyond conferring stress-responsiveness to genes not directly bound by ATF4, XBP1, or ATF6, this expansion also allows for transcriptional suppression, to the extent that ATF4, XBP1, or ATF6 enhance the expression of repressive transcription factors.

The best-characterized example of a secondary UPR-regulated transcription factor is C/EBP Homologous Protein (CHOP). CHOP is a direct target of ATF4 and ATF6 (Ma et al., 2002), and its translation is stimulated by eIF2α phosphorylation (Palam et al., 2011). Phosphorylation of CHOP by p38 MAP kinase appears to be required for its full activity (Wang and Ron, 1996). A member of the C/EBP family of transcriptional regulators, CHOP can form heterodimers with other C/EBP proteins, and was proposed to act as a dominant-negative inhibitor of C/EBPα and β in particular (Ron and Habener, 1992). It is now clear that CHOP possesses both activating and repressing potential, and so its effect on the transcriptome is complex.

CHOP is strongly functionally associated with cell death; both cells and animals lacking CHOP are protected from a diverse array of stressful stimuli (Zinszner et al., 1998; Oyadomari and Mori, 2004). Due at least in part to the very short half-life of both the protein and its mRNA, CHOP expression is strongly correlated with the ER stress burden in real-time (Rutkowski et al., 2006). Hence, the window of time in which CHOP can exert a direct effect on the transcriptome is tightly controlled, as one might expect *a priori* for a factor that potentiates cell death. As a transcriptional regulator rather than a conventional pro-apoptotic effector, CHOP promotes cell death through the regulation of several classes of downstream genes. CHOP has been proposed to regulate the expression of both anti-apoptotic and pro-apoptotic genes of the *Bcl2* family (McCullough et al., 2001; Puthalakath et al., 2007). In addition, CHOP regulates expression of the bZIP factor ATF5, which is itself translationally regulated by eIF2α phosphorylation (Watatani et al., 2008; Zhou et al., 2008), and the targets of ATF5 include the pro-apoptotic protein NOXA (Teske et al., 2013). However, ChIP-seq analysis revealed that its direct targets are most prominently enriched for genes involved in protein synthesis, which are co-regulated by ATF4 (Han et al., 2013). Among these genes is GADD34, which indicates that CHOP controls a negative feedback loop allowing for the dephosphorylation of eIF2α and resumption of protein synthesis, even if the ER is ill-equipped to handle nascent protein influx (Marciniak et al., 2004). A consequence of restored protein synthesis is increased production of reactive oxygen species (ROS), likely resulting from oxidative protein folding in the ER; indeed, CHOP also regulates expression of the ER oxidase ERO1α (Li et al., 2009), indicating that CHOP promotes oxidative folding in tandem with increased ER influx. Blunting either ROS accumulation or protein synthesis neuters CHOP's pro-apoptotic potential (Marciniak et al., 2004; Malhotra et al., 2008; Li et al., 2009).

The strong association of CHOP with cell death in both cell and animal models raises the question of whether CHOP is intrinsically apoptotic, or instead whether this role is only manifested in the context of severe stress, masking other roles for CHOP in maintaining normal physiologic homeostasis. Indeed, the restoration of protein synthesis following stress is essential to maintain vital cellular functions, and the existence of a constitutive phosphatase ensures that even *Chop*^−/−^ or *Gadd34*^−/−^ cells are able to resume protein synthesis (Harding et al., 2009; Tsaytler et al., 2011); CHOP, therefore, merely accelerates the process, and it is possible that the kinds of stresses encountered in normal (i.e., non-pathologic) physiology are sufficiently mild that the benefits to cellular function of restoring protein synthesis outweigh the cost of increased ROS production. In addition, while CHOP induction is largely suppressed in some instances of physiological UPR induction such as B lymphocyte differentiation (Gass et al., 2002) and toll-like receptor ligation (Woo et al., 2009), it occurs in others such as feeding after a fast (Pfaffenbach et al., 2010).

CHOP likely contributes directly to the suppression of several metabolic transcriptional regulators during ER stress in the liver (Chikka et al., 2013). This finding suggests that CHOP might serve a role in regulating lipid metabolism *in vivo*, which is consistent with the steatosis observed in *Chop*^−/−^ mice (Maris et al., 2012). To the extent that CHOP (or other secondary stress-regulated transcription factors) regulates metabolic transcription factors such as *Cebpa*, *Ppara*, and *Srebf1c*, there exists a multistep gene regulatory network during ER stress in hepatocytes that culminates in changes in the expression of genes encoding rate-limiting enzymes of intermediary metabolism (Rutkowski et al., 2008). CHOP also promotes inflammation (Maris et al., 2012; DeZwaan-McCabe et al., 2013; Malhi et al., 2013), although whether this is a consequence of direct CHOP action on inflammatory genes, or instead a secondary consequence of the other functions of CHOP is not yet clear.

The actions of CHOP on the transcriptome and on the accompanying physiological processes highlight the ability of the UPR to expand its reach through the regulation of secondary transcription factor expression, but CHOP is certainly not the only transcription factor whose transcription is regulated by ER stress. In fact, a search through several published microarrays from ER stress-treated MEFs (Marciniak et al., 2004; Wu et al., 2007; Rutkowski et al., 2008) reveals expression changes for several dozen transcription factors and cofactors, including both activators and repressors (Table 1). It follows that such transcriptional cascading will create a temporal hierarchy of gene regulation, with the earliest regulated genes being most proximally connected to UPR pathways. Such cascading likely contributes to the regulation of metabolic genes in the liver [(Arensdorf et al., 2013) *this issue*].

**Table 1:** **Transcription factors and cofactors whose mRNA expression is regulated by the UPR^a^**.

**Likely activator**	**Likely repressor**	**Both activities demonstrated**
**UPREGULATED**		
*Aatf*	*Cry1*	*Atf2*
*Arnt1*	*Cry2*	*Atf3*
*Atf4*	*Hey2*	*Cebpg*
*Atf6*	*Mybbp1a*	*Ddit3 (Chop)*
*Ets2*	*Sin3a*	*Myc*
*Fubp1*	*Zfp57*	*Nfil3*
*Hoxa1*		*Rbpj*
*Hoxa11*		
*Myst4*		
*Nfya*		
*Nr4a2*		
*Rxrb*		
*Snip1*		
*Tfcp2*		
*Zbtb7b*		
**DOWNREGULATED**		
*Foxq1*	*Id1*	*E2f8*
*Nfkbiz*	*Nr1d1*	*Elk3*
	*Nfkbia*	*Hipk2*
		*Stat3*

a 1.5-fold or more, p <0.05, in at least 2 of the arrays described in Marciniak et al. (2004); Wu et al. (2007); and Rutkowski et al. (2008).

### Heteromeric interactions and cofactor titration

The major transcription factors of the UPR, both canonical and secondary, are bZIPs (ATF4, ATF6, XBP1, CHOP, JUN, ATF3, ATF5). The dozens of members of this family form homotypic and heterotypic dimers, typically within functionally related subclasses (Vinson et al., 2006). Thus, the complement of genes regulated during ER stress can be influenced by the formation of novel regulatory complexes not possible when the UPR is inactive, containing one UPR-regulated member and one constitutively expressed member. For instance, as a C/EBP family member, CHOP can interact with C/EBPα and C/EBPβ, altering the transactivation potential of each of these (Fawcett et al., 1996; Ubeda et al., 1996; Chiribau et al., 2010). Likewise, ATF6 was recently shown to interact with C/EBPβ upon stimulation with the inflammatory cytokine IFN-γ to transcriptionally upregulate the autophagy-promoting gene *Dapk1* (Gade et al., 2012).

Stress-regulated transcription factors can also influence global gene expression beyond the genes they directly regulate through the titration of coregulatory molecules shared with constitutive transcription factors. Such a mechanism was demonstrated recently for the coactivating factor CRTC2, which was shown to lose its costimulatory interaction with the gluconeogenic bZIP transcription factor CREB in favor of an interaction with ATF6 (Wang et al., 2009). The consequence of this titration was inhibition of hepatic gluconeogenesis during acute ER stress, and gluconeogenesis and hyperglycemia could be suppressed in diabetic animals by ATF6 overexpression.

ATF6 has also been shown to interact with the coactivator PGC-1α. In skeletal muscle, this interaction promoted the full upregulation of canonical UPR target genes upon exercise (Wu et al., 2011). Likewise, a PGC-1α/ATF6 interaction stimulated expression of the ERRγ orphan nuclear receptor/transcription factor in a hepatocyte cell line (Misra et al., 2013). Roles for ERRγ in glucose and alcohol metabolism in the liver have recently emerged (Kim et al., 2012, 2013). PGC-1α has been implicated in the transcriptional regulation of many key metabolic processes including gluconeogenesis, fatty acid oxidation, and mitochondrial biogenesis, based on its ability to coactivate a number of transcription factors (Lin et al., 2005). Therefore, it is possible that ER stress will disrupt or otherwise influence the regulation of such gene networks based on competition for PGC-1α binding, although this has not yet been demonstrated.

### Expansion of UPR signaling pathways

The status of PERK, IRE1, and ATF6 as canonical UPR regulators arises from the primacy of their early discoveries and their ubiquity. However, a number of other stress-signaling pathways have since been discovered that extend the scope of the UPR in both general and context-specific ways.

While IRE1α is ubiquitously expressed, its paralog IRE1β, expressed in mucin-producing cells of the gut (Bertolotti et al., 2001) and airway (Martino et al., 2013), was identified around the same time (Wang et al., 1998). Like its paralog, IRE1β can catalyze the splicing of *Xbp1* (Calfon et al., 2002). Mice lacking IRE1β are sensitive to colitis induced by dextran sodium sulfate challenge (Bertolotti et al., 2001), as are animals lacking XBP1 (Kaser et al., 2008), ATF6, or the ER cochaperone p58^*IPK*^/ERDJ6 (Cao et al., 2013). The phenotypic similarities among these animals suggests that, like its paralog, IRE1β contributes to ER homeostasis largely through *Xbp1* splicing and upregulation of the ER folding and quality control machinery. However, the endonuclease domain of IRE1β displays less activity toward *Xbp1* than does that of IRE1α and has an enhanced specificity for *28s* rRNA, which can contribute to suppression of protein synthesis (Imagawa et al., 2008). In addition, in contrast to *Ire1α*^−/−^ animals, *Ire1β*^−/−^ mice showed elevated expression of *mucin 2* mRNA, impaired MUC2 secretion, and exacerbated ER stress, including, paradoxically, increased *Xbp1* splicing (Tsuru et al., 2013). These findings suggest that IRE1α and β have at least partially separable functions, and raise the question of how strong a contribution IRE1β makes to mRNA regulation in the cells where it is expressed.

IRE1β aside, the reach of the UPR has been most expanded by the discovery of ER localized proteins that, like ATF6, are activated by regulated intramembrane proteolysis. First among these was a paralog of ATF6 known as ATF6β (ATF6 is also known as ATF6α) that is 36 percent identical to ATF6 over 93 percent of its length (Haze et al., 2001). Cells lacking ATF6β show no apparent defect in upregulation of canonical UPR target genes (Yamamoto et al., 2007). However, like ATF6, ATF6β binds to ERSE sequences in conjunction with NFY (Yoshida et al., 2001a). Mice lacking both ATF6 and ATF6β die during embryogenesis (Yamamoto et al., 2007), as do similarly manipulated medaka fish (Ishikawa et al., 2012). Overexpression of *Bip* could partially rescue impaired notochord development in these fish, suggesting that ATF6 and ATF6β converge on chaperone mRNA regulation, albeit with somewhat different kinetics (Haze et al., 2001). Whether ATF6β regulates the expression of any unique genes is not yet known.

In addition to ATF6 and ATF6β, there are at least 5 additional ER-resident transmembrane bZIP transcription factors that are cleaved by RIP, including CREBH, Luman, OASIS, BBF2H7, and CREB4 [reviewed in (Asada et al., 2011)]. These 5 proteins are not highly homologous to each other or to ATF6, and each is expressed in a unique subset of tissues, but they are all known or thought to be cleaved by S1P (Raggo et al., 2002; Murakami et al., 2006; Stirling and O'Hare, 2006; Zhang et al., 2006). These proteins can be activated by conventional ER stress and/or can regulate the expression of UPR target genes through traditional ER stress-responsive *cis*-acting sequences (Kondo et al., 2005; Liang et al., 2006b; Stirling and O'Hare, 2006; Zhang et al., 2006; Kondo et al., 2007). However, they may be activated more strongly by physiological signals that influence ER homeostasis in ways other than the simple perturbation of or excess demand upon the protein folding machinery, and might also be retained in the ER by distinct mechanisms; at least CREBH appears to be retained by virtue of its cytosolic membrane-proximal segment rather than by lumenal *Bip* binding as for ATF6 (Llarena et al., 2010). In addition, they also appear to regulate expression of distinct sets of genes, suggesting that they diversify the responsiveness of the UPR and the scope of genes that it regulates in various tissues rather than simply augmenting these processes.

The predominantly liver-specific RIP substrate CREBH illustrates the complex relationship between these substrates and the canonical UPR. *Crebh* mRNA is upregulated by conventional ER stress in the liver, and cleavage of the protein is modestly stimulated as well; the cleaved form is capable of associating with ATF6 and upregulating *UPRE*- or *ERSE*-dependent reporters (Zhang et al., 2006). However, both its expression and cleavage are also strongly induced by inflammatory stimuli such as LPS exposure or IL-6 treatment, and the genes encoding the inflammatory modulators CRP and SAP were also identified as likely CREBH targets. While inflammatory stimuli such as LPS appear capable of inducing ER stress, they spawn a modified eIF2α-independent response (Woo et al., 2009), suggesting that, even if CREBH is activated simply by the accumulation of unfolded proteins in the ER, it would be so in the context of a modified UPR. CREBH also regulates the expression of hepicidin, leading to dysregulation of iron homeostasis upon ER stress in wild-type mice but not *Crebh*^−/−^ animals (Vecchi et al., 2009). Therefore, CREBH can also contribute to the expansion of mRNA expression even during exposure to a conventional ER stressor. More recently, CREBH was shown to directly regulate genes involved in gluconeogenesis (Lee et al., 2010) and lipid metabolism (Zhang et al., 2012). The latter of these processes showed a stronger CREBH dependence in the context of a high-fat atherogenic diet, raising the question of the extent to which physiological stimuli such as obesity impact gene expression through the canonical UPR vs. through pathways that enlist the action of molecules like CREBH to produce a unique response.

Knockout of each of the RIP substrates [or, in the case of Luman, a regulator of the pathway, LRF; (Martyn et al., 2012)] yields a discrete phenotype attributable to dysfunction of the major tissue in which the substrate is expressed (Asada et al., 2011). In contrast, mice lacking ATF6, which have compromised ER chaperone induction upon ER stress, show no apparent basal phenotype (Wu et al., 2007; Yamamoto et al., 2007). Therefore, simple failure to upregulate ER chaperones to the maximum extent is not sufficient to elicit a phenotype, making it unlikely that the RIP substrates merely augment chaperone induction; more likely, the phenotypes induced by their deletion are attributable to non-redundant actions on specific genes, be they chaperones or something else entirely. However, their activation signals and the global effects on transcriptome regulation remain unclear.

## mRNA stability

Although each UPR pathway culminates in production of a transcription factor, mRNA abundance can just as readily be regulated by enhanced or diminished stability. Indeed, one estimate of the relative contribution of transcriptional and post-transcriptional mechanisms to mRNA abundance during ER stress—based on the comparison of mRNA levels in nuclear run-off assays against total mRNA pools—suggested that ~75 percent of mRNAs were regulated at least in part at the level of stability (Kawai et al., 2004). Pathways linking ER stress to mRNA stability are much less understood than transcriptional mechanisms, but are emerging as important influences on UPR output and physiological responses.

### Gene regulation by RIDD

A subset of ER-localized mRNAs are degraded directly by the endonuclease activity of IRE1 in a process called regulated IRE1-dependent decay (RIDD). The process was first observed in *Drosophila* cells, in which a subset of mRNAs was rapidly suppressed by ER stress in an IRE1α-dependent but XBP1-independent manner (Hollien and Weissman, 2006). This group of mRNAs was highly enriched for those encoding proteins with in-frame ER targeting sequences, but the cleavage of these mRNAs was otherwise non-sequence-specific. It is possible that RIDD represents an ancient activity of IRE1, since Ire1p in the fission yeast *S. pombe* catalyzes a RIDD activity but not a *HAC1* splicing activity (Kimmig et al., 2012). More recently, a sequence necessary for mRNA cleavage by IRE1α *in vitro* was identified (Oikawa et al., 2010), although it is not yet clear whether these findings extend to RIDD targets *in vivo* as well. These findings led to the idea that activated IRE1α directly cleaves some mRNAs that are brought to proximity with the ER membrane by virtue of their association with translating ribosomes synthesizing signal peptide- or signal anchor-encoding proteins. This finding was subsequently extended to mammalian cells (Han et al., 2009; Hollien et al., 2009). The RIDD and *Xbp1* splicing activities of IRE1α are functionally separable (Han et al., 2009; Hollien et al., 2009), implying that each process plays a distinct role in UPR-mediated control over the transcriptome. Where these functions overlap—i.e., in the expression of ER chaperones and other ER-localized proteins that facilitate recovery from stress but whose mRNAs are RIDD targets by virtue of their localization—the transcriptional induction must be sufficient to overcome degradation by RIDD.

Although the RIDD pathway has not yet been as well characterized in mammalian cells, its targets in that context are involved in processes ranging from signaling cascades (e.g., *Pdgfrb*, *Efnb2*, *Ncam1*, *Raptor*) to transcription (e.g., *Pbxip1*, *Hoxb4*, *Srsf3*) to lysosomal degradation (e.g., *Bloc1s1*, *Tpp1*, *Hgsnat*) to xenobiotic metabolism (Cytochrome p450s-encoding genes) and energy production (e.g., *Oxct1*) (Hollien et al., 2009; Hur et al., 2012). RIDD appears now not to be solely limited to genes encoding ER-translocated proteins but includes mRNAs encoding cytosolic factors as well (Hollien et al., 2009; Oikawa et al., 2010; Ventoso et al., 2012). This ability of the ER-tethered IRE1α to degrade mRNAs encoding cytosolic proteins might arise from the localization of specific cytosolic mRNAs in the process of translation to the ER membrane (Stephens et al., 2005); indeed, localization of an mRNA to the ER membrane, irrespective of whether it encodes a protein of the endomembrane system, appears sufficient to target that mRNA for RIDD for the large majority of mRNAs, at least in insect cells (Gaddam et al., 2012). In addition, even mRNAs not stably associated with the ER membrane can still be targeted for RIDD if they contain an Xbp1-like stem-loop structure that allows them to associate directly with IRE1 (Moore et al., 2013).

Irrespective of whether RIDD acts on specific mRNAs or more generally on most of those associated with the ER membrane, its activation has distinct physiological consequences. IRE1β (but not α) might control efflux of absorbed lipids through its RIDD activity. Lipids absorbed from the diet are packaged by enterocytes into lipoprotein particles known as chylomicrons, and a key step in this packaging is lipidation of apolipoproteins in the ER by microsomal triglyceride transfer protein (MTTP) (Hussain, 2000). IRE1β was found to cleave *Mttp* mRNA, and *Ire1β*^−/−^ mice on a high fat diet had elevated MTTP expression, elevated chylomicron production, and hyperlipidemia (Iqbal et al., 2008). The RIDD pathway also can regulate lipid metabolism in the liver; IRE1α-dependent degradation of genes involved in lipogenesis and lipoprotein synthesis was elicited as a feedback mechanism in mice lacking XBP1 (So et al., 2012). An analogous pathway of feedback elicited IRE1α-dependent suppression of proinsulin processing in pancreatic β cells of *Xbp1*^−/−^ mice (Lee et al., 2011). These findings are consistent with the idea that RIDD is most active on ER-localized mRNAs, since lipoprotein formation, lipogenesis, and proinsulin processing all take place in the ER or at the ER membrane. A conservative estimate puts the frequency of RIDD targets at ~5 percent of all ER stress-regulated mRNAs in cultured mammalian cells (Hollien et al., 2009), and it might in fact be substantially higher (Gaddam et al., 2012). Whether RIDD is more or less active during physiological stimuli *in vivo* and whether its specificity for certain groups of substrates can be meaningfully regulated are not yet understood.

### mRNA regulation by miRNAS

Another common source of mRNA regulation occurs through microRNAs (miRNAs). miRNAs are short (~22 nt) single-stranded RNAs which bind to complementary mRNAs and promote their degradation or, less frequently, inhibit their translation (Valencia-Sanchez et al., 2006). The UPR-mediated regulation of miRNA is a rapidly emerging area of investigation and a potential mechanism for fine-tuning of mRNA abundance. The pathways leading from UPR activation to miRNA regulation and the consequences of this regulation for mRNA abundance and downstream cellular processes have been reviewed extensively in (Maurel and Chevet, 2013), to which we direct the reader for details. Each of the three canonical UPR pathways transcriptionally regulates the expression of discrete miRNAs (Bartoszewski et al., 2011; Belmont et al., 2012; Byrd et al., 2012; Chitnis et al., 2012; Gupta et al., 2012). To date, the best-described functions of ER stress-regulated miRNAs are in tuning UPR sensitivity (Byrd et al., 2012; Maurel et al., 2013; Zhang et al., 2013) or in regulating cell proliferation and apoptosis during stress (Chitnis et al., 2012; Duan et al., 2012; Gupta et al., 2012; Muratsu-Ikeda et al., 2012).

As an emerging area of study, the physiological roles of ER stress-mediated miRNA regulation are largely unknown; however, it was recently demonstrated that IRE1α activation causes degradation of miR-17, which in turn leads to upregulation of the miR-17 target mRNA encoding thioredoxin-interacting protein (TXNIP) (Lerner et al., 2012). TXNIP promoted inflammasome assembly, caspase-1 activation, and cell death, and *Txnip*^−/−^ mice were protected from pancreatic β cell death induced by production of misfolded insulin. Given the ability of miRNAs to coordinately regulate the stability of many target mRNAs, it seems likely that this mechanism will emerge as a major contributor to noncanonical UPR output, and that further physiological roles will be discovered.

ER stress has been associated with changes in the stability of individual mRNAs through undetermined mechanisms (Pereira et al., 2010; Park et al., 2012), and so it is possible that other pathways for regulating mRNA turnover exist as well. One suggested mechanism is the sequestration of translationally inhibited mRNAs in stress granules (Kimball et al., 2003). mRNA stabilization through sequestration would thus decouple mRNA abundance from protein abundance (Kawai et al., 2004). This possibility illustrates the caution that must be exercised when interpreting changes in mRNA abundance in general: mRNA and protein expression are only loosely correlated (Gygi et al., 1999), and understanding the mechanisms of mRNA regulation by the UPR only illuminates one component in the regulation of gene expression.

## A way forward: dissecting UPR-responsive gene regulatory networks

The abundance of non-canonical mechanisms of mRNA regulation by the UPR suggest that the number of mRNAs whose regulation is attributable to direct binding by XBP1, ATF4, or ATF6 is likely to represent only a small portion of all the regulated genes. How, then, can the complex gene regulatory network of the UPR be best studied moving forward? The explosion of high throughput methodology and increasingly sophisticated bioinformatic tools holds promise for both “top-down” and “bottom-up” approaches to this problem. The application of microarray technology to the yeast UPR first revealed the complexity of the transcriptional response (Travers et al., 2000); similar microarray-based approaches (Harding et al., 2003; Lee et al., 2003b; Wu et al., 2007) in mammals revealed the dependencies of subsets of genes on each UPR pathway, but could not separate direct from indirect influences. Next-generation sequencing methodologies, including mRNA-seq and ChIP-seq, will now be used to piece together regulatory hierarchies; these techniques were recently combined to elucidate the gene networks regulated by XBP1 (Acosta-Alvear et al., 2007) and CHOP and ATF4 (Han et al., 2013). A complementary approach will be to find groups of genes that are coordinately regulated and use bioinformatic analysis to predict previously hidden upstream regulators. As proof-of-principle, we have shown that the temporal organization of metabolic gene regulation upon ER stress in the liver identifies the transcription factor HNF4α as a key link between UPR activation and the expression of genes involved in lipid metabolism [(Arensdorf et al., 2013); this issue]. Such functional genomics approaches have until recently been restricted to studies in simple organisms like yeast. However, the ability to probe and experimentally manipulate the entire mammalian genome has now made these techniques feasible in higher eukaryotes as well (Kampmann et al., 2013), and these approaches have been used to understand secretory pathway function (Bassik et al., 2013) and the ERAD network (Christianson et al., 2011).

Although best known as the gateway to the secretory pathway, the ER participates in many cellular processes that have little or nothing to do with protein folding *per se*. While augmentation of the ER protein folding capacity is certainly a significant consequence of UPR activation, it remains to be seen whether most of the genes regulated by the UPR ultimately redound to this capacity, or whether the UPR has been co-opted in the homeostatic regulation of other cellular processes—particularly those, such as lipid metabolism, that involve the ER. Deciphering the pathways leading from UPR activation to mRNA regulation will allow the functional significance of non-canonical UPR signaling mechanisms to be understood in the contexts of normal and pathological physiology.

### Conflict of interest statement

The authors declare that the research was conducted in the absence of any commercial or financial relationships that could be construed as a potential conflict of interest.

## Supplementary material

The Supplementary Material for this article can be found online at: http://www.frontiersin.org/journal/10.3389/fgene.2013.00256/abstract
