# Supplementary material for: Regulation of the transcriptome by ER stress: non-canonical mechanisms and physiological consequences
Source: Front Genet. 2013 Dec 2;4:256. doi: 10.3389/fgene.2013.00256 (PMC3844873; doi:10.3389/fgene.2013.00256)
Supplement: Supplementary file 1 [file DataSheet1.PDF]

Supplementary Material

Computational Model

To create a simple computational model of transcriptional regulation, a system of two nonlinear ordinary differential equations was created. The first describes the upstream regulating transcription factor “A4”, modeled loosely off of the expression pattern of ATF4 in response to treatment of MEFs with 2.5 nM thapsigargin. The equation describing the downstream target “c” allows “A4” to either stimulate or inhibit transcription or degradation of “c”, using the α or β parameter, respectively. The other parameters for this equation were chosen to elicit similar maximal extents of regulation (~10-fold) for each manipulation.

Equations

A4 (transcription factor):

$$\frac{dA4}{dt} = ate^{-bt^N} \tag{1}$$

c (mRNA):

$$\frac{dc}{dt} = k_s\left(1 + \alpha\frac{A4^n}{A4^n + K_s^n}\right) - k_d\left(1 + \beta\frac{A4}{A4 + K_d}\right)c \tag{2}$$

Parameters

TF equation (1)

$a = 0.0916,\ b = 3.86 \times 10^{-6},\ N = 2$

These parameters were chosen to fit the data for the level of ATF4 during the UPR in WT cells treated with 2.5 nM TG.

mRNA equation (2)

$k_s = k_d = 0.00144\ (min^{-1})$ for stable mRNA (long half-life)

$k_s = k_d = 0.00393\ (min^{-1})$ for unstable mRNA (short half-life)

$K_s = K_d = 5$ = Affinity constant (high affinity; at peak expression of A4, the stimulation/inhibition is 80% of its saturation level)

$n = 1$ = Hill coefficient (no cooperativity)

α is the parameter for the degree to which the TF stimulates or inhibits the transcription of mRNA (chop). $\alpha < 0$ means the TF inhibits transcription, $\alpha = 0$ means no influence, and $\alpha > 0$ means the TF stimulates transcription.

β is the parameter for the degree to which the TF stimulates or inhibits the degradation of mRNA (chop). $\beta < 0$ means the TF inhibits degradation, $\beta < 0$ means no influence, and $\beta < 0$ means the TF stimulates degradation.

Simulations

A4 stimulates transcription *(α = 18.6* for long half-life; α = *13.0* for short half-life; *β = 0)*

A4 inhibits transcription *(α = -1.85* for long half-life; α = *-1.29* for short half-life; *β = 0)*

A4 stimulates degradation *(α = 0; β = 10)*

A4 inhibits degradation *(α = 0; β = -3* for long half-life; β = *-1.97* for short half-life*)*
